# A case report of atypical hemolytic uremic syndrome with a CFH mutation complicated by recurrent posterior reversible encephalopathy syndrome

**DOI:** 10.1097/MD.0000000000049593

**Published:** 2026-07-03

**Authors:** Ryotaro Maruyama, Daisuke Takeuchi, Masashi Momose, Tetsuro Amemiya, Ion Terakawa, Kenichi Teshima, Jun Chimura, Kyutaro Noju, Ryu Sugimoto, Kosuke Shiroto, Kazuki Suganuma, Tomokiyo Yamamoto

**Affiliations:** a Department of General Internal Medicine, Aizawa Hospital, Nagano, Japan; b Department of Emergency and Critical Care Medicine, Aizawa Hospital, Nagano, Japan.

**Keywords:** atypical hemolytic uremic syndrome, complement inhibition, complement-mediated thrombotic microangiopathy, posterior reversible encephalopathy syndrome

## Abstract

**Rationale:**

Atypical hemolytic uremic syndrome (aHUS) is a rare thrombotic microangiopathy (TMA) characterized by microangiopathic hemolytic anemia, thrombocytopenia, and organ dysfunction, particularly affecting the kidneys and the central nervous system. It is caused by genetic variants or autoantibodies involved in dysregulation of the complement system. Pathogenic variants of the complement factor H (CFH) gene are associated with severe disease and may lead to life-threatening multiorgan failure during the acute phase. We report a case of CFH mutation-positive aHUS complicated by recurrent posterior reversible encephalopathy syndrome (PRES) during the course of treatment.

**Patient concerns:**

A 32-year-old woman presented with progressive fatigue and acute dyspnea. Laboratory tests revealed hemolytic anemia, thrombocytopenia, and severe acute kidney injury.

**Diagnoses:**

A peripheral blood smear revealed schistocytes, consistent with TMA. On hospital day 1, she developed generalized tonic–clonic seizures followed by respiratory arrest that required mechanical ventilation. Continuous hemodialysis was initiated for severe renal failure. After the exclusion of thrombotic thrombocytopenic purpura, typical hemolytic uremic syndrome, and secondary causes of TMA, a clinical diagnosis of aHUS was made. Genetic testing later revealed a pathogenic heterozygous CFH variant (c.3572C>T, p.Ser1191Leu), confirming the diagnosis of aHUS.

**Interventions:**

Anti-C5 monoclonal antibody (eculizumab) was initiated based on the clinical diagnosis of aHUS. Intensive care management, including mechanical ventilation, renal replacement therapy, plasma exchange, and strict blood pressure control, was also provided.

**Outcomes:**

During the course of the disease, she developed severe cardiomyopathy and recurrent PRES. Early initiation of complement inhibition combined with intensive care management resulted in complete neurological recovery, and the patient was discharged home without any sequelae.

**Lessons:**

This case highlights that CFH mutation-positive aHUS can be complicated by severe multiorgan dysfunction, including cardiomyopathy and recurrent PRES. Early clinical recognition and prompt initiation of complement inhibition were central to the favorable outcome, while intensive blood pressure control and comprehensive supportive care may have contributed to neurological recovery.

## 1. Introduction

Atypical hemolytic uremic syndrome (aHUS), a complement-mediated form of thrombotic microangiopathy (TMA), is a subtype of TMA caused by the dysregulation of the alternative complement pathway. It is typically characterized by a triad of microangiopathic hemolytic anemia, thrombocytopenia, and organ dysfunction, particularly involving the kidneys and the central nervous system. Genetic variants that affect complement regulatory proteins or the presence of specific autoantibodies play a central role in disease pathogenesis.

Here, we report a case of a 32-year-old woman with CFH mutation-positive aHUS who developed recurrent posterior reversible encephalopathy syndrome (PRES) during the course of treatment. This case highlights the importance of early diagnosis and appropriate therapeutic intervention in aHUS and suggests a relationship between neurological complications and hypertension.

## 2. Case presentation

A 32-year-old previously healthy woman with no family history of hematologic or renal disease presented with a 2-month history of progressive fatigue and anorexia. Her symptoms worsened following a COVID-19 infection 1 month prior to admission, resulting in impaired activities of daily living. One day before admission, she developed dyspnea at rest and was referred to our hospital after laboratory tests at a previous facility revealed anemia, thrombocytopenia, and severe acute kidney injury.

She had no significant past medical history other than dysmenorrhea. On arrival, her vital signs were as follows: body temperature, 35.7°C; blood pressure, 162/97 mm Hg; heart rate, 104 beats/min; respiratory rate, 24 breaths/min; and oxygen saturation, 100% on room air. Physical examination revealed generalized pitting edema, conjunctival pallor, and purpura of the trunk and extremities.

Laboratory findings showed hemoglobin, 5.1 g/dL; platelet count, 87,000/μL; lactate dehydrogenase, 623 U/L; blood urea nitrogen, 158.5 mg/dL; and serum creatinine, 22.72 mg/dL. Urinalysis revealed proteinuria (4+), hematuria (3+), and waxy casts (1+), consistent with severe renal impairment. A peripheral blood smear revealed schistocytes exceeding 5% of the red blood cells, strongly suggesting TMA. The PLASMIC score was 5, indicating an intermediate risk for thrombotic thrombocytopenic purpura (TTP). She was immediately admitted to the intensive care unit, where comprehensive supportive care was initiated.

On the night of day 1 of hospitalization, she experienced a generalized tonic–clonic seizure followed by respiratory arrest, necessitating mechanical ventilation and vasoactive medications. Continuous hemodialysis was initiated for severe acute kidney injury. Diagnostic investigations included measurement of ADAMTS13 activity, stool cultures, autoimmune-related antibodies (including antinuclear antibodies and antineutrophil cytoplasmic antibodies), vitamin B12 levels, and pregnancy testing.

Plasma exchange therapy was initiated on hospital day 3. She was extubated on the same day; however, she subsequently developed acute cardiomyopathy with worsening hypoxemia that required reintubation. Transthoracic echocardiography revealed basal hyperkinesis with severe apical hypokinesis, a reduced left ventricular ejection fraction of 30%, and an elevated tricuspid regurgitation pressure gradient (60.9 mm Hg), suggesting significant cardiac dysfunction.

On hospital day 6, involuntary eye movements were observed. Head computed tomography revealed widespread hypodense lesions predominantly involving the occipital lobes, consistent with severe PRES. Intensive blood pressure control was initiated. A diagnostic workup showed ADAMTS13 activity of 56%, negative stool cultures, negative autoimmune antibodies, elevated vitamin B12 levels, and a negative pregnancy test, making TTP, typical HUS, and secondary TMAs unlikely. On the basis of these findings, the patient was clinically diagnosed with aHUS (Table 1).

**Table 1:** Laboratory findings at admission.

Test	Result	Unit	Test	Result	Unit
WBC	5450	/μL	Anti-nuclear antibody (ANA)	<40	Titer
Hb	5.1	g/dL	Complement C3	85	mg/dL
Plt	8.7	×10^4^/μL	Complement C4	26	mg/dL
MCV	87.6	Fl	CH50	41.2	U/mL
MCHC	32.7	%	ADAMTS13 activity	56	%
T-bli	0.5	mg/dL	ADAMTS13 inhibitor	<0.5	BU/mL
AST	13	U/L	Anti-ARS antibody	<5.0	Index
ALT	6	U/L	Anti-cardiolipin β2-glycoprotein I antibody	<1.2	U/mL
ALP	38	U/L	Anti-cardiolipin IgG antibody	<4.0	U/mL
LDH	623	U/L	PR3-ANCA	<1.0	U/mL
γ-GT	16	U/L	Anti-GBM antibody	<2.0	U/mL
CK	125	U/L	IgG	842	mg/dL
BUN	158.5	mg/dL	IgA	198	mg/dL
Cre	22.72	mg/dL	IgM	95	mg/dL
Na	135	mEq/L	Vitamin B12	1500	pg/mL
K	5.4	mEq/L	Folate	22.0	ng/mL
Cl	101	mEq/L	Haptoglobin	10	mg/dL
Ca	7.8	mg/dL	SCT ratio	0.63	–
NT-proBNP	77311	pg/dL	Homocysteine	8.8	nmol/mL
CRP	0.17	mg/dL	Direct Coombs test	Negative	–
PT-INR	1.03	INR	Urinary hCG	Negative	–
APTT	27.5	sec	Immunoelectrophoresis	M-bow negative	–
Fib	321.9	mg/dL	Serum protein electrophoresis	M-peak negative	–
D-dimer	13.3	μg/dL			
FDP	25.3	μg/dL			

Hematologic parameters, renal function, immunological markers, and complement-related biomarkers at presentation are summarized.

ADAMTS13 = a disintegrin and metalloproteinase with thrombospondin type 1 motif, member 13, ALP = alkaline phosphatase, ALT = alanine aminotransferase, ANA = antinuclear antibody, APTT = activated partial thromboplastin time, ARS = aminoacyl-tRNA synthetase, AST = aspartate aminotransferase, BUN = blood urea nitrogen, CH50 = 50% hemolytic complement activity, CK = creatine kinase, Cre = serum creatinine, CRP = C-reactive protein, FDP = fibrin/fibrinogen degradation products, Fib = fibrinogen, GBM = glomerular basement membrane, Hb = hemoglobin, hCG = human chorionic gonadotropin, LDH = lactate dehydrogenase, MCHC = mean corpuscular hemoglobin concentration, MCV = mean corpuscular volume, Plt = platelet count, PR3-ANCA = proteinase 3-antineutrophil cytoplasmic antibody, PT-INR = prothrombin time-international normalized ratio, SCT = silica clotting time, T-bil = total bilirubin, WBC = white blood cell count, γ-GT = gamma-glutamyl transferase.

Given the severity of the acute kidney injury and the presence of life-threatening neurological complications, eculizumab was initiated on hospital day 6 based on a clinical diagnosis rather than awaiting genetic confirmation. Following the initiation of eculizumab, meningococcal vaccination was promptly administered, and prophylactic ceftriaxone was continued for 2 weeks.

**Figure 1.**
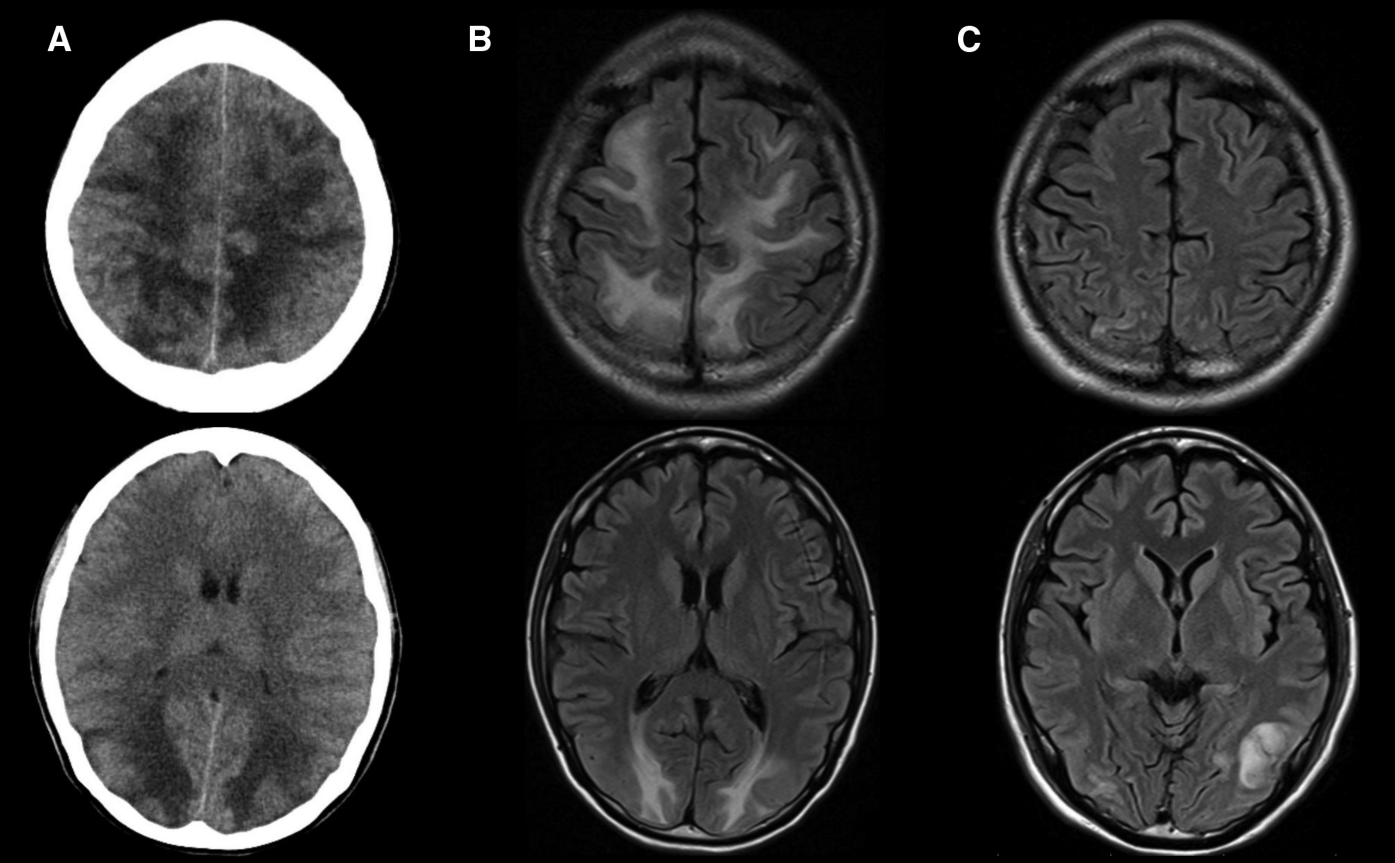
Serial neuroimaging findings during the clinical course. (A) Head computed tomography performed on hospital day 6 demonstrated widespread hypodense lesions involving the occipital and parietal lobes. (B) Brain magnetic resonance imaging (MRI) performed on hospital day 14 revealed hyperintense lesions in the same regions on fluid-attenuated inversion recovery (FLAIR) images. (C) Follow-up brain MRI on hospital day 23 demonstrated newly developed hyperintense lesions in the left temporal lobe, distinct from the previously observed lesions.

The patient was weaned from mechanical ventilation on hospital day 13 and discharged from the intensive care unit on day 14. Brain magnetic resonance imaging on day 14 revealed extensive fluid attenuated inversion recovery hyperintense lesions, predominantly in the occipital lobes. On hospital day 23, a renal biopsy was performed to assess renal prognosis. On the same day, she experienced an abrupt second generalized tonic–clonic seizure, and brain magnetic resonance imaging revealed new fluid attenuated inversion recovery hyperintense lesions in regions different from those previously observed, suggestive of recurrent PRES (Fig. 1).

Although the reduction in schistocytes was delayed, gradual hematologic improvement was achieved with combination therapy using eculizumab and plasma exchange, which was discontinued on hospital day 31 following clinical stabilization.

**Figure 2.**
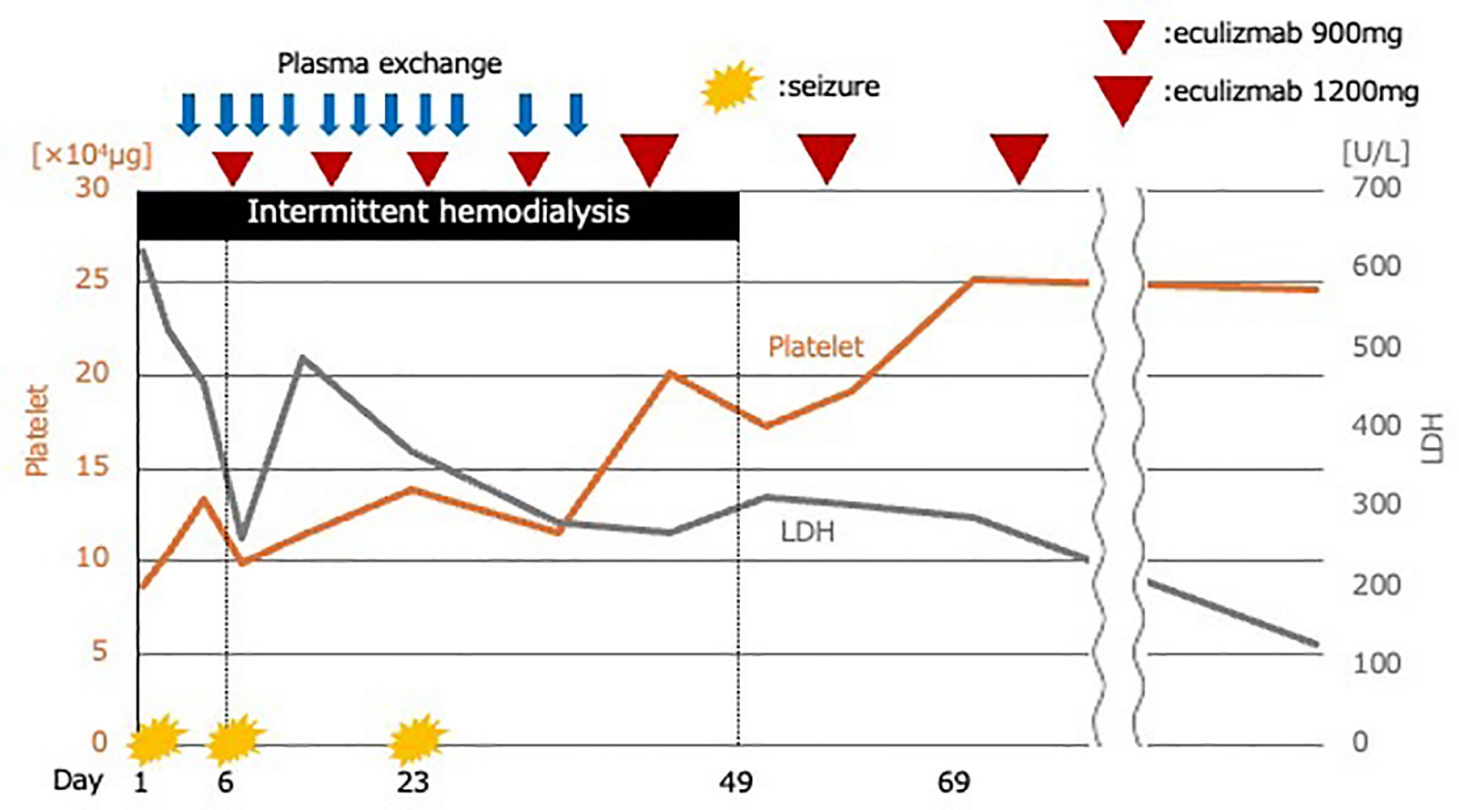
Summary of the clinical course and therapeutic interventions. Major clinical events, neuroimaging findings, laboratory trends, and treatments are presented according to hospital day.

Thereafter, anemia and thrombocytopenia gradually improved, and urine output progressively increased. Genetic testing on hospital day 38 revealed a pathogenic heterozygous CFH variant (c.3572C>T, p.Ser1191Leu), which confirmed the diagnosis of aHUS. She was weaned off intermittent hemodialysis on hospital day 49. Eculizumab therapy was continued, and she was discharged home on hospital day 69 with no neurological deficits (modified Rankin Scale score, 0). The patient is currently receiving maintenance therapy with ravulizumab in an outpatient setting (Fig. 2).

## 3. Discussion

This case provides important clinical insights into the intensive care management of CFH mutation-associated aHUS. First, this case illustrates that aHUS may present with severe multiorgan dysfunction, including cardiomyopathy and recurrent PRES, requiring intensive care management. Recurrent PRES in the context of aHUS has rarely been reported,^[1–5]^ suggesting that persistent complement dysregulation and hypertension may contribute to repeated cerebrovascular endothelial injury.

The complement system is activated through 3 pathways: classical, lectin, and alternative pathways. Unlike the other 2 pathways, the alternative pathway lacks a specific recognition molecule and is continuously activated at a low level, making it particularly vulnerable to dysregulation.^[6]^ Abnormal activation of this pathway plays a central role in aHUS pathogenesis. Complement-related abnormalities are broadly classified into loss-of-function variants, which affect regulatory proteins and gain-of-function variants affecting complement activators. Loss-of-function variants have been reported in CFH, CFI, CD46, VTN, and THBD, whereas gain-of-function variants of CFB and C3 result in excessive complement activation. Additionally, acquired aHUS caused by anti-factor H antibodies has been reported, indicating an autoimmune mechanism in a subset of patients.

Our patient carried a pathogenic loss-of-function CFH variant (c.3572C>T, p.Ser1191Leu), which has been associated with severe disease and poor renal outcomes.^[7,8]^ This variant has been previously reported to be pathogenic in aHUS and is located in the C-terminal short consensus repeat domains (SCR19–20), which are essential for binding to C3b and host cell surfaces and protecting endothelial cells from complement-mediated injury.^[9]^ Japanese registry data show that approximately 30% of patients with aHUS progress to death or end-stage kidney disease.^[7]^ Nationwide studies in Japan reported 118 cases between 1998 and 2016 and 209 cases between 2014 and 2019, highlighting the rarity of this condition in clinical practice.^[8,10]^

Second, this case emphasizes the importance of early complement inhibition based on clinical diagnosis rather than waiting for genetic confirmation. Genetic testing and anti-factor H antibody assays require substantial time, and approximately 30% to 40% of patients have no identifiable genetic variants.^[11]^ Delayed initiation of complement inhibition has been associated with irreversible organ damage in previous studies.^[10,12–15]^ In this case, early recognition of TMA and prompt exclusion of TTP enabled timely initiation of eculizumab, which likely contributed to favorable neurological and renal outcomes.

aHUS frequently causes extrarenal complications, among which neurological involvement is the most common, occurring in approximately 10%s to 48% of patients. Extrarenal manifestations occur in approximately 20% of cases, and severe multiorgan failure is observed in approximately 5%.^[16]^ Our patient developed severe neurological complications, including encephalopathy and acute symptomatic seizures, as well as cardiovascular complications, including cardiomyopathy and heart failure, which necessitated mechanical ventilation and vasoactive medications. While several case reports have described aHUS complicated by PRES, to our knowledge, recurrent PRES has rarely been documented.^[1–5]^ Notably, previously reported cases of aHUS complicated by PRES, including the present case, have generally shown favorable neurological recovery.

Finally, this case highlights the potential importance of intensive blood pressure control in preventing neurological complications in patients with TMA. Previous studies have demonstrated a correlation between elevated blood pressure and neurological manifestations regardless of TMA subtype.^[17]^ Persistent endothelial injury due to complement dysregulation together with uncontrolled hypertension may increase the risk of PRES recurrence, highlighting the importance of intensive blood pressure management in the acute phase.

This study has several limitations. As a single case report, the causal relationships between complement dysregulation, hypertension, and PRES recurrence cannot be definitively established. However, this case emphasizes the importance of early clinical diagnosis, prompt complement inhibition, and intensive hemodynamic and blood pressure management in patients with severe aHUS.

## 4. Conclusion

We report a case of CFH mutation-positive aHUS complicated by severe multiorgan dysfunction, including cardiomyopathy and recurrent PRES, requiring intensive care management with mechanical ventilation and renal replacement therapy. Early clinical diagnosis and prompt initiation of complement inhibition, combined with intensive blood pressure control, resulted in complete neurological recovery and favorable outcomes. This case suggests that early clinical recognition and prompt initiation of complement inhibition are central to favorable outcomes, while intensive blood pressure control and comprehensive supportive care may have contributed to neurological recovery.

## Acknowledgments

We would like to thank the staff of the Department of Nephrology for providing hemodialysis and plasma exchange to the patient.

## Author contributions

**Investigation:** Masashi Momose, Tetsuro Amemiya, Ion Terakawa, Kenichi Teshima, Jun Chimura, Kyutaro Noju, Ryu Sugimoto, Kosuke Shiroto, Kazuki Suganuma.

**Supervision:** Daisuke Takeuchi, Tomokiyo Yamamoto.

**Writing – original draft:** Ryotaro Maruyama.

**Writing – review & editing:** Ryotaro Maruyama.

## References

[1] Medeni SS Namdaroglu S Cetintepe T. An adult case of atypical hemolytic uremic syndrome presented with posterior reversible encephalopathy syndrome: successful response to late-onset eculizumab treatment. Hematol Rep. 2018;10:7553. 30344987 10.4081/hr.2018.7553 PMC6176395

[2] Povey H Vundru R Junglee N Jibani M. Renal recovery with eculizumab in atypical hemolytic uremic syndrome following prolonged dialysis. Clin Nephrol. 2014;82:326–31. 23557793 10.5414/CN107958

[3] Muralidharan S Mathew GG Alwan A Jayaprakash V. A rare case of atypical hemolytic uremic syndrome presenting as chronic interstitial nephritis. Cureus. 2024;16:e65274. 39184759 10.7759/cureus.65274 PMC11343015

[4] Sajan T Vinay S Sonu N Alan P. How atypical can atypical hemolytic uremic syndrome be? Clin Case Rep. 2014;2:57–9. 25356245 10.1002/ccr3.59 PMC4184631

[5] Okabe M Kobayashi A Marumoto H. Renal damage in recurrent atypical hemolytic uremic syndrome associated with C3 p.Ile1157Thr gene mutation. Intern Med. 2021;60:917–22. 33087669 10.2169/internalmedicine.5716-20 PMC8024950

[6] Harboe M Mollnes TE. The alternative complement pathway revisited. J Cell Mol Med. 2008;12:1074–84. 18419792 10.1111/j.1582-4934.2008.00350.x PMC3865650

[7] Fakhouri F Roumenina LT Provot F. Genetics and outcome of atypical hemolytic uremic syndrome: a nationwide French series comparing children and adults. Clin J Am Soc Nephrol. 2010;5:1740–9. 10.2215/CJN.04760512 PMC3613948 23307876

[8] Fujisawa M Kato H Yoshida Y. Clinical characteristics and genetic backgrounds of Japanese patients with atypical hemolytic uremic syndrome. Clin Exp Nephrol. 2018;22:1088–99. 29511899 10.1007/s10157-018-1549-3 PMC6437120

[9] Ersoy Dursun F Yesil G Sasak G Dursin H. Familial atypical hemolytic uremic syndrome with positive p.S1191L (c.3572C>T) mutation on the CFH gene: a single-center experience. Balkan J Med Genet. 2021;24:81–8. 34447663 10.2478/bjmg-2021-0007 PMC8366473

[10] Tatematsu Y Imaizumi T Michihata N. Annual trends in atypical haemolytic uremic syndrome management in Japan and factors influencing early diagnosis and treatment: a retrospective study. Sci Rep. 2024;14:18265. 39107421 10.1038/s41598-024-68736-6 PMC11303750

[11] Alasfar S Alachkar N. Atypical hemolytic uremic syndrome post-kidney transplantation: two case reports and review of the literature. Front Med (Lausanne). 2014;1:52. 25593925 10.3389/fmed.2014.00052 PMC4292050

[12] Japanese Society of Nephrology, Japanese Society for Dialysis Therapy, Japanese Society of Pediatric Nephrology. Clinical practice guideline for atypical hemolytic uremic syndrome (aHUS) 2023. Japanese Society of Nephrology; 2023.

[13] Legendre CM Licht C Muus P. Terminal complement inhibitor eculizumab in atypical hemolytic-uremic syndrome. N Engl J Med. 2013;368:2169–81. 23738544 10.1056/NEJMoa1208981

[14] Fakhouri F Schwotzer N Frémeaux-Bacchi V. How I diagnose and treat atypical hemolytic uremic syndrome. Blood. 2023;141:984–95. 36322940 10.1182/blood.2022017860

[15] Walle JV Delmas Y Ardissino G Wang J Kincaid JF Haller H. Improved renal recovery in patients with atypical hemolytic uremic syndrome following rapid initiation of eculizumab treatment. J Nephrol. 2017;30:127–34. 26995002 10.1007/s40620-016-0288-3 PMC5316393

[16] Raina R Krishnappa V Blaha T. Atypical hemolytic-uremic syndrome: an update on pathophysiology, diagnosis, and treatment. Ther Apher Dial. 2019;23:4–21. 30294946 10.1111/1744-9987.12763

[17] Halimi JM Thoreau B von Tokarski F. What is the impact of blood pressure on neurological symptoms and the risk of ESKD in primary and secondary thrombotic microangiopathies based on clinical presentation: a retrospective study. BMC Nephrol. 2022;23:39. 35057750 10.1186/s12882-022-02672-3 PMC8781095

